# Computer-Mediated Communication in Adults With and Without Moderate-to-Severe Traumatic Brain Injury: Survey of Social Media Use

**DOI:** 10.2196/26586

**Published:** 2021-08-27

**Authors:** Emily L Morrow, Fangyun Zhao, Lyn Turkstra, Catalina Toma, Bilge Mutlu, Melissa C Duff

**Affiliations:** 1 Department of Hearing & Speech Sciences Vanderbilt University Medical Center Nashville, TN United States; 2 Department of Psychology University of Wisconsin-Madison Madison, WI United States; 3 Department of Computer Sciences University of Wisconsin-Madison Madison, WI United States; 4 Speech-Language Pathology Program, School of Rehabilitation Science McMaster University Hamilton, ON Canada; 5 Department of Communication Arts University of Wisconsin-Madison Madison, WI United States

**Keywords:** traumatic brain injury, social media, disability, rehabilitation, cognitive communication

## Abstract

**Background:**

Individuals with a history of traumatic brain injury (TBI) report fewer social contacts, less social participation, and more social isolation than noninjured peers. Cognitive-communication disabilities may prevent individuals with TBI from accessing the opportunities for social connection afforded by computer-mediated communication, as individuals with TBI report lower overall usage of social media than noninjured peers and substantial challenges with accessibility and usability. Although adaptations for individuals with motor and sensory impairments exist to support social media use, there have been no parallel advances to support individuals with cognitive disabilities, such as those exhibited by some people with TBI. In this study, we take a preliminary step in the development process by learning more about patterns of social media use in individuals with TBI as well as their input and priorities for developing social media adaptations.

**Objective:**

This study aims to characterize how and why adults with TBI use social media and computer-mediated communication platforms, to evaluate changes in computer-mediated communication after brain injury, and to elicit suggestions from individuals with TBI to improve access to social media after injury.

**Methods:**

We conducted a web-based survey of 53 individuals with a chronic history of moderate-to-severe TBI and a demographically matched group of 51 noninjured comparison peers.

**Results:**

More than 90% of participants in both groups had an account on at least one computer-mediated communication platform, with Facebook and Facebook Messenger being the most popular platforms in both groups. Participants with and without a history of TBI reported that they use Facebook more passively than actively and reported that they most frequently maintain web-based relationships with close friends and family members. However, participants with TBI reported less frequently than noninjured comparison participants that they use synchronous videoconferencing platforms, are connected with acquaintances on the web, or use social media as a gateway for offline social connection (eg, to find events). Of the participants with TBI, 23% (12/53) reported a change in their patterns of social media use caused by brain injury and listed concerns about accessibility, safety, and usability as major barriers.

**Conclusions:**

Although individuals with TBI maintain social media accounts to the same extent as healthy comparisons, some may not use them in a way that promotes social connection. Thus, it is important to design social media adaptations that address the needs and priorities of individuals with TBI, so they can also reap the benefits of social connectedness offered by these platforms. By considering computer-mediated communication as part of individuals’ broader social health, we may be able to increase web-based participation in a way that is meaningful, positive, and beneficial to broader social life.

## Introduction

### Computer-Mediated Communication and Social Participation

Social media and other computer-mediated communication (CMC) platforms are ubiquitous parts of everyday life and have radically altered how we work, live, and build and maintain social networks. CMC includes any form of web-based communication, which may be synchronous (eg, video conferencing platforms) or asynchronous (eg, web-based messaging) and may involve the exchange of text, audio, or video messages for professional or social purposes. Within this realm, more than 3 billion people worldwide use social media platforms, such as Facebook, Twitter, and Instagram [1]. Users on social media may participate actively by broadcasting personal or nonpersonal information and providing feedback on others’ posts, or they may participate passively by observing information posted by others [2-5]. Depending on how they use social media, individuals may derive different benefits. For example, social media users may derive greater social capital (or value from web-based relationships) if they use social media for active communication, have a diverse web-based network, and increase web-based social connectedness [6].

### Tailored Social Media Adaptations to Increase Access for Individuals With Disabilities

For many individuals with disabilities, social media platforms have the potential to overcome existing barriers to social participation [7]. For example, individuals with reduced or limited mobility may be able to engage in social activities where in-person attendance is prohibitive. There have also been increasing calls to foster social media accessibility for those with sensory differences. For instance, it has become more common to add alternative text to images on social media platforms to reduce participation barriers for individuals with certain visual disabilities [8]. These efforts have allowed many individuals with motor and sensory disabilities to increase participation in this ubiquitous part of daily modern social life.

### Traumatic Brain Injury and Expanding Social Media Adaptation for Cognitive Disabilities

Adolescents and adults with traumatic brain injury (TBI) often report being socially isolated [9] and could benefit from social media participation. However, many of these individuals have cognitive disabilities that may affect social media use. Individuals with TBI may experience changes in memory, social perception, and social communication [10] and thus may find it more challenging to perceive text-based social cues in social media than cues present in face-to-face communication [11,12]. TBI-related memory and learning disabilities may make it difficult to keep up with evolving requirements of regularly updating social media platforms [3], and reduced attention may create challenges parsing critical information from cluttered news feeds [13].

Tailored adaptations may allow individuals with cognitive disabilities to access and benefit from social media more easily. However, adaptations for individuals with motor and sensory disabilities have not been paralleled by advances that address barriers to social media participation for individuals with *cognitive* disabilities [3,14], such as those experienced by many individuals with TBI. Given that participation in social media platforms is a critical part of day-to-day social communication for many adults, increasing access to CMC and social media platforms may hold significant promise for increasing overall social participation for individuals with TBI [2,3,13].

It is critical to understand how TBI-related cognitive and communication challenges affect access to and use of social media, so we can design apps that support access for all. For example, TBI may affect overall access, such that individuals with TBI are less likely to use social media than noninjured peers. Alternatively, it may be that individuals with TBI use social media at similar rates to noninjured peers but do not reap the same social benefits because of challenges with cognition and communication. Thus, the first step in developing tools that will increase social media success in individuals with TBI is to gather more information about how individuals with TBI use social media and how brain injury affects social media use.

### Social Media Use After TBI

There is an emerging body of research directed at understanding social media use in individuals with TBI. Perhaps consistent with the fact that current social media platforms are not designed with individuals with cognitive disabilities in mind, adults in the chronic phase of TBI report using social media less frequently than noninjured peers [3] and indicate that they face significant challenges with accessibility and usability [15,16]. Baker-Sparr et al [3] surveyed a large cohort of individuals with chronic TBI (n=337) on whether and how they use the internet and found that although the proportion of internet users with TBI was high (250/337, 74.1%), it was significantly lower than general population estimates of internet usage (84%) [17]. In this survey, 14.8% (37/250) of individuals with TBI reported that brain injury had somewhat affected their ability to use the internet, listing memory problems, visual challenges, and difficulty with attention as barriers [3].

Consistent with these responses, other work by Ketchum et al [18] has shown a positive association between social internet use and in-person social participation in individuals with TBI, suggesting that individuals with TBI are not likely to use social media as an alternative to social communication. Instead, they may face many of the same barriers and facilitators on the web as they do in person.

Brunner et al [13] interviewed 13 adults in the chronic phase after an acquired brain injury (traumatic or nontraumatic) about their use of social media. Facebook was the most popular social media platform among interviewees (consistent with Baker-Sparr et al [3]), followed by Twitter and Instagram. Most participants reported that they had help setting up their social media accounts and that they use social media more than once a day. All participants stated that they were motivated to use social media to stay connected with others, and some (2/13, 15% of interviewees) reported using social media to help other people with brain injury. Some participants reported feeling overwhelmed or confused by social media, and those who felt confused by a given platform were likely to give up using it [13].

Our study extends previous work in several ways. First, we examined patterns of social media use in adults with TBI and a demographically matched comparison group. The use of a matched comparison group allows us to understand how patterns may differ between individuals with and without a history of TBI who are similar in demographic variables that may affect CMC use (eg, age and education). Second, we asked specific questions about *how* usage has changed as a result of TBI, rather than just *if* usage has changed, and solicit direct suggestions for improving social media technology support for individuals with TBI. Asking individuals with TBI for specific input on improving social media participation aligns with national and international priorities for TBI rehabilitation research that includes patient-reported outcomes to support health and independence [19]. This study is, to our knowledge, the first in-depth survey of social media use of individuals with TBI and matched peers in the United States and has a larger sample size than previous interview-based studies, allowing us to combine breadth and depth in understanding patterns of social media use after brain injury. Together, these study characteristics increase experimental rigor and expand our knowledge on how individuals with TBI use social media, if and how their use of social media has changed following their brain injury, and the nature of the barriers they face.

### Study Objectives

This study had three specific aims: (1) to characterize how and why adults with TBI use social media and CMC platforms, (2) to evaluate changes in CMC after brain injury, and (3) to elicit suggestions from individuals with TBI to improve access to social media after injury.

## Methods

### Participants

Participants (or respondents) were 53 individuals with moderate-to-severe TBI (28 women) and 51 noninjured comparison (NC) participants (29 women). All participants were recruited from Nashville, Tennessee, United States, and the surrounding areas, and the groups were demographically matched for age and education. The mean age was 37.7 years (SD 9.6) for the TBI group and 36.4 years (SD 10.4) for the NC group, with no significant between-group difference (*t*_102_=0.685; *P=.*50). The mean years of education were 15.0 (SD 2.6) for respondents with TBI and 15.1 (SD 2.1) for the NC group, with no significant between-group difference (*t*_102_=0.581; *P*=.56).

Individuals with TBI were recruited through the Vanderbilt Brain Injury Patient Registry and had no self-reported preinjury history of neurological or cognitive disability. All individuals with TBI were in the chronic phase of injury (>6 months postonset, mean time since onset 74.1 months, SD 66.0) and sustained their injuries as adults. Injury-related information was obtained from medical records and semistructured participant interviews. Injury etiologies included motor vehicle accidents (n=27), falls (n=10), being struck by a vehicle as a pedestrian (n=4), motorcycle or snowmobile accidents (n=3), nonmotorized vehicle accidents (eg, biking, n=3), assault (n=3), and being struck by a moving object (n=3). TBI severity was determined using the Mayo Classification System [20], so injuries were moderate-to-severe if at least one of the following criteria were met: (1) Glasgow Coma Scale score <13 within 24 hours of acute care admission, (2) positive neuroimaging findings (acute computed tomography findings or lesions visible on chronic magnetic resonance imaging), (3) loss of consciousness >30 minutes, or (4) posttraumatic amnesia >24 hours.

NC participants were recruited from the NC participant pool in the Vanderbilt Brain Injury Patient Registry and had no self-reported history of neurological or cognitive disability.

### Survey

The data reported here are part of a larger survey examining different aspects of social media use for individuals with TBI. For all participants, the survey included questions about social media platform use, activities on social media, types and quality of relationships with social media friends, and perceived benefits and drawbacks of using social media. Participants with TBI also answered questions about how social media use has changed since injury, provided suggestions for researchers and clinicians interested in decreasing barriers and supporting social media use, and responded to mockups of potential Facebook modifications.

In designing the survey, we designated some questions to focus specifically on Facebook usage because we anticipated high usage of Facebook across both groups based on national data [1] and previous work on social media in TBI [3,13]. We sought to acquire additional information about how and why individuals with TBI use Facebook to guide the future design of technology-based aids around the Facebook platform, given its high usage among individuals with TBI.

Here, we focus on questions related to our goals in characterizing social media use in TBI, assessing postinjury differences in CMC, and describing barriers to social media use for individuals with TBI. We included items from the Social Networking Usage Questionnaire [21] to assess participants’ activities on social media. We also included questions from an analysis of Facebook friend networks [22] to evaluate participants’ web-based social networks and from an assessment of web-based social capital formation [6] to assess how individuals with and without TBI use social media in passive and active ways. Items from these scales are presented in the *Results* section. Some of these items were modified for participants with TBI. For example, we added TBI-related examples to tailor questions about social media advocacy and groups. In addition, we collapsed response options on the web-based social capital formation scale [6] from five items (almost never, rarely, sometimes, almost every day, and multiple times a day) to three items (never or almost never, sometimes, and often) to reduce the cognitive load on respondents, given the survey’s overall length. As we did not intend to directly compare these responses with results from previous studies, modifying established scales maintained a connection to how social media use is studied by field experts while remaining feasible for participants with TBI.

### Procedures

The procedures for this study were approved by the Human Research Protections Program at Vanderbilt University. Participants received a link to complete the survey on the web via REDCap (Research Electronic Data Capture) [23]. All participants completed the survey between June and September 2020. The survey consisted of up to 280 questions (a mix of multiple-choice and free response), but in this study, we focus on 15 questions relevant to social media usage and changes in usage related to TBI (Multimedia Appendix 1). In some cases, questions only appeared if respondents had previously selected a given response. For example, individuals only described their participation frequency for the social media platforms they reported using. The survey took approximately 30 to 45 minutes for the NC participants to complete. As participants with TBI responded to more questions, they received links to take the survey in two parts, lasting approximately 30 minutes each.

### Analysis and Interpretation

The goal of this study was to explore how and why individuals with and without a history of TBI use social media and CMC platforms as well as existing barriers to social media use for individuals with TBI. Consistent with this exploratory goal, we used descriptive statistics, expecting that the data would serve as the foundation for future hypothesis-driven research on technology-based social media interventions for individuals with TBI [24].

The survey provided several opportunities to add information via free-text responses to open-ended questions, and we have reported these responses descriptively. Although it is important to not overinterpret those responses (as we did not understand the forces that caused only some individuals to respond to these questions), we included them because they could generate and refine questions for future research [24].

## Results

### Survey Overview

Responding to individual questions was voluntary, and some questions only appeared via branching logic, depending on previous responses. Therefore, not all participants answered all questions. The number of individuals who responded to a given question is listed in parentheses. Response percentages for each group are listed for multiple-choice questions; proportions are not listed for free-text responses. On some questions, respondents could choose more than one option, so the total percentages reported for those questions may exceed 100%.

### CMC Platforms and Frequency of Use

Overall, 96% (51/53) of participants with TBI and 98% (50/51) of NC participants reported that they currently hold an account on at least one CMC platform. Participants frequently used more than one platform, with participants with TBI holding an account on an average of 5.66 (SD 3.26) platforms and NC participants holding an account on an average of 7.49 (SD 3.57) platforms.

Table 1 provides the proportion of participants in each group, reporting that they use certain platforms. Facebook was the most popular platform for both groups (TBI: 41/51, 80%; NC: 43/50, 86%). Facebook Messenger came next for participants with TBI, followed by Instagram, FaceTime, and Snapchat. Twitter was popular with NC participants (23/50, 46%), although it was not used by as many participants with TBI (13/51, 26%). Participants with TBI reported lower rates of usage for LinkedIn (TBI: 19/51, 37%; NC: 29/50, 58%) and Pinterest (TBI: 16/51, 31%; NC: 23/50, 46%) as well as other videoconferencing platforms, such as Zoom (TBI: 19/51, 37%; NC: 31/50, 62%) and Skype (TBI: 13/51, 26%; NC: 24/50, 48%).

Participants could also include, in free-text form, the names of platforms they use that were not on the survey list. A total of five platforms were reported: GroupMe, Amazon Show, WebEx for participants with TBI, and Kik and Slack for NC participants.

We asked the participants to indicate how often they used platforms where they had an account. Table 2 provides the frequency of use of platforms used by at least 25% of participants with TBI. The full table, including all platforms, is available in Multimedia Appendix 1.

**Table 1:** Proportion of participants who endorsed having an account on a given social media platform.

Platform	Traumatic brain injury (n=51), n (%)	Noninjured comparison (n=50), n (%)
Bumble	7 (14)	1 (2)
Discord	4 (8)	5 (10)
Facebook	41 (80)	43 (86)
Facebook Messenger	38 (75)	40 (80)
FaceTime	25 (49)	28 (56)
Google Hangouts	7 (14)	13 (26)
Hinge	4 (8)	4 (8)
Instagram	30 (59)	37 (74)
LINE	0 (0)	0 (0)
LinkedIn	19 (37)	29 (58)
Pinterest	16 (31)	23 (46)
Quora	0 (0)	3 (6)
Reddit	8 (16)	11 (22)
Skype	13 (26)	24 (48)
Snapchat	25 (49)	23 (46)
Telegram	1 (2)	1 (2)
TikTok	3 (6)	9 (18)
Tinder	6 (12)	4 (8)
Tumblr	3 (6)	7 (14)
Twitter	13 (26)	23 (46)
Viber	0 (0)	2 (4)
WhatsApp	15 (29)	18 (36)
Zoom	19 (37)	31 (62)
Other platform	3 (6)	3 (6)

**Table 2:** Frequency of use for respondents on a given platform.

Platform^a^	Yearly (%)			Monthly (%)			Weekly (%)			Multiple times per week (%)			Daily (%)			Multiple times per day (%)	
	TBI^b^	NC^c^	TBI		NC	TBI		NC	TBI		NC	TBI		NC	TBI		NC
Facebook (TBI: n=41; NC: n=43)	0	5	2		9	12		2	10		16	30		21	46		47
Facebook Messenger (TBI: n=38; NC: n=40)	5	10	8		18	16		20	26		28	24		12	21		12
FaceTime (TBI: n=25; NC: n=28)	0	11	48		29	12		36	28		21	8		0	4		3
Instagram (TBI: n=30; NC: n=37)	7	3	17		5	10		3	7		11	13		32	46		46
LinkedIn (TBI: n=19; NC: n=29)	26	17	16		44	32		24	10		7	16		4	0		4
Pinterest (TBI: n=16; NC: n=23)	31	26	25		35	13		13	19		17	6		0	6		9
Skype (TBI: n=13; NC: n=23)	46	30	31		35	0		9	8		4	15		13	0		9
Snapchat (TBI: n=25; NC: n=23)	8	17	16		0	16		22	20		17	12		17	28		27
Twitter (TBI: n=13; NC: n=22)	23	14	15		27	15		14	23		9	9		14	15		22
WhatsApp (TBI: n=15; NC: n=18)	33	39	33		27	20		11	0		6	14		6	0		11
Zoom (TBI: n=19; NC: n=31)	11	3	31		10	21		29	11		29	15		19	11		10

^a^This table includes platforms where at least 25% of participants with traumatic brain injury endorsed having an account.

^b^TBI: traumatic brain injury.

^c^NC: noninjured comparison.

### Reasons for Not Using Social Media

We asked participants if there were any social media platforms where they would like to have an account but do not currently. A total of 4% (2/52) of participants with TBI reported wanting to use LinkedIn, TikTok, and Tinder. A total of 8% (4/51) of NC participants reported that they would like to have accounts on Facebook, Reddit, Snapchat, TikTok, Tumblr, and Twitter. In a separate question, we asked participants to explain why they did not use these platforms. The participant with TBI who responded to this question stated, “I don’t like the way I communicate on them. I don’t like the way I obsess over how often I am on them.” A total of 2 NC participants also noted that time is a factor in not setting up additional social media accounts.

We asked participants who do not have a Facebook account to explain via free text why they do not use the platform. Participants with TBI stated that they were concerned about the nature of information available on Facebook (eg, falsehoods, n=3) or that they found Facebook to be “toxic” or “superficial*”* (n=2). One participant with TBI stated a preference for in-person communication, “I like eye contact, and raw emotion, not emojis.” The most frequent reason NC participants gave for not using Facebook was that they found it unnecessary or a waste of time (n=3).

### Activities on Social Media

We asked participants about how they use social media with options adapted from the Social Networking Usage Questionnaire [21]. Table 3 provides social media activities endorsed by participants with TBI and NC participants. Participants with TBI reported numerically less frequently than NC participants that they use social media to keep up with friends and family (TBI: 44/52, 85%; NC: 49/50, 98%) or to obtain information regarding social events (ie, to use CMC for relationships and events happening beyond social media; TBI: 24/52, 46%; NC: 33/50, 66%). Participants with TBI also reported numerically less frequently than NC participants that they use social media to obtain information (eg, to discover new things, TBI: 26/52, 50%; NC: 36/50, 72%; to follow current events, TBI: 25/52, 48%; NC: 28/50, 56%) or to post about their daily lives (TBI: 19/52, 37%; NC: 25/50, 50%).

**Table 3:** Percentage of participants endorsing different uses of social media (adapted from a study by Gupta and Bashir [21]).

Type of use	TBI^a^ (n=52), n (%)	Noninjured comparison (n=50), n (%)
Advocating for specific causes (eg, promoting TBI-related organizations or events)	8 (15)	14 (28)
Creating my social identity	11 (21)	17 (34)
Discovering new things	26 (50)	36 (72)
Following thought leaders or celebrities	7 (14)	21 (42)
Getting information regarding social events	24 (46)	33 (66)
Getting job-related information	19 (37)	20 (40)
Keeping in touch with friends and family	44 (85)	49 (98)
Looking for support groups	4 (8)	4 (8)
Providing support to others	10 (19)	15 (30)
Searching for specific information (eg, information about TBI)	11 (21)	17 (34)
Staying up to date with news and current events	25 (48)	28 (56)
Sharing the happenings of daily life	19 (37)	25 (50)
Sharing new ideas	10 (19)	7 (14)

^a^TBI: traumatic brain injury.

For those individuals who reported having a Facebook account (42 participants with TBI and 43 NC participants), we asked questions about their specific use of the platform. First, we asked them what kinds of friends they have on Facebook (options adapted from a study by Manago et al [22]; Table 4). Both groups most frequently reported being Facebook friends with close friends and family members. Participants with TBI reported numerically less frequently than NC participants that they were Facebook friends with acquaintances, coworkers, people they had met once, or people they casually dated.

**Table 4:** Types of Facebook friends endorsed by participants (adapted from a study by Manago et al [22]).

Type of Facebook friend	Traumatic brain injury (n=42), n (%)	Noninjured comparison (n=43), n (%)
Acquaintance	33 (79)	41 (95)
Band, musical artist, or other celebrity	14 (33)	18 (42)
Best friend	36 (86)	35 (81)
Classmate	33 (79)	33 (77)
Coworker	32 (76)	37 (86)
Current significant other (eg, girlfriend or boyfriend)	28 (67)	21 (49)
Family member	38 (91)	39 (91)
Fellow club member	7 (17)	16 (37)
Fraternityor sorority brother or sister	6 (14)	5 (12)
Friend of a friend	27 (64)	29 (67)
Good friend	38 (91)	38 (88)
High school friend	35 (83)	39 (91)
Neighbor	20 (48)	16 (37)
Web-based friend only (never met in person)	11 (26)	14 (33)
Past romantic partner	20 (48)	24 (56)
Roommate	16 (38)	17 (40)
Someone you do not know	10 (24)	12 (28)
Someone you casually dated	14 (33)	22 (51)
Someone you met in a different country	12 (29)	18 (42)
Someone you only met once	19 (45)	30 (70)
Teammate	14 (33)	15 (35)
Very good friend	36 (86)	36 (84)

We asked participants questions from a scale on social capital building [6] to assess how they use Facebook actively (eg, creating their own posts), passively (eg, looking through the newsfeed), for social searching (eg, actively searching for and adding friends) and browsing (eg, looking at others’ profiles but not adding them as friends), and for private communication (Table 5). Participants with and without a history of TBI reported more frequently that they use Facebook passively than actively. Although not by large numerical differences, participants with TBI reported more frequently, in general, than NC participants that they use Facebook for social browsing (eg, browsing through others’ profiles) and social searching (eg, looking for new friends to add).

**Table 5:** Facebook users’ social capital building activities (adapted from a study by Koroleva et al [6]).

Activity		Never or almost never, n (%)			Sometimes, n (%)			Often, n (%)	
		TBI^a^ (n=42)	NC^b^ (n=43)	TBI (n=42)		NC (n=43)	TBI (n=42)		NC (n=43)
**Active participation**									
	Post something	14 (33)	18 (42)	23 (55)		18 (42)	5 (12)		7 (16)
	Share thoughts and feelings	26 (62)	28 (65)	14 (33)		13 (30)	2 (5)		2 (5)
	Share something you are interested in	14 (33)	18 (42)	21 (50)		20 (47)	7 (17)		5 (12)
	Share your impressions with your friends	24 (57)	24 (56)	13 (31)		14 (33)	5 (12)		5 (12)
	Donate to a cause on Facebook	30 (71)	31 (72)	10 (24)		12 (28)	2 (5)		0 (0)
**Passive following**									
	Follow your friends’ news	10 (24)	7 (16)	18 (43)		13 (30)	14 (33)		23 (54)
	Look through your newsfeed	5 (12)^c^	2 (5)	15 (37)^c^		13 (30)	21 (51)^c^		28 (65)
	Click on content shared by friends	9 (21)	3 (7)	17 (41)		25 (58)	16 (38)		15 (35)
**Social browsing**									
	Browse your friends’ profiles	13 (31)	7 (16)	19 (45)		29 (67)	10 (24)		7 (16)
	Browse through friends of your friends	23 (55)	22 (51)	12 (29)		16 (37)	7 (17)		5 (12)
	Look at profiles of people not on your Facebook friends list	21 (50)	27 (63)	15 (36)		12 (28)	6 (14)		4 (9)
**Social searching**									
	Search for people to add	26 (62)	31 (72)	12 (29)		11 (26)	4 (10)		1 (2)
	Send friendship requests	17 (42)^c^	19 (44)	20 (49)^c^		22 (51)	4 (10)^c^		2 (5)
	Add people suggested by Facebook	24 (57)	26 (61)	14 (33)		16 (37)	4 (10)		1 (2)
**Private communication**									
	Send private messages	10 (24)	13 (30)	23 (55)		22 (51)	9 (21)		8 (19)
	Chat	16 (38)	19 (44)	20 (48)		20 (47)	6 (14)		3 (9)

^a^TBI: traumatic brain injury.

^b^NC: noninjured comparison.

^c^n=41.

### Reflections on CMC Use After TBI

We asked participants with TBI whether their use of social media has changed because of the injury, and 23% (12/53) responded affirmatively. Next, we asked 12 participants to describe the changes via free text. A total of 5 participants reported that they spend more time on social media than before their injuries, whereas 2 participants reported spending less. A total of 2 participants stated that they now use social media to keep up with TBI-related groups. Another 2 participants endorsed being more careful in their use of social media (ie, whom they follow). Some participants stated that using social media has become harder postinjury (because of sensitivity to light or screens: n=1; increased stress: n=1; or feelings of insecurity: n=1), but one participant noted that social media is helpful in managing a memory deficit.

We also asked participants with TBI to provide suggestions for researchers and clinicians interested in improving the experience of using social media for individuals with TBI, and 32% (17/53) of participants provided free-text suggestions. Some participants worried that social media may be detrimental for people with TBI (n=3) or noted that clinicians should discourage overuse (n=2). In contrast, other participants (n=2) noted that social media may be helpful for individuals with TBI, particularly for learning and social interactions that feel less stressful than face-to-face communication. Other participants suggested reducing “extra content” (eg, advertisements and recommended posts, n=2), which can feel overwhelming, or increasing provider presence on social media (eg, via support groups, n=2).

## Discussion

### Principal Findings

#### Overview

The primary goal of this survey was to understand the patterns of social media use among individuals with TBI. We compared their social media usage with peers without a history of TBI, solicited feedback on how brain injury changes social media use, and requested suggestions as to how best to support individuals with TBI in their social media use. Several key observations have emerged.

#### Variability in Social Media Use for Adults With and Without TBI

Social communication differences are a hallmark of the observed cognitive disability in TBI [25]. Adults with TBI report fewer social contacts, less social participation, and more social isolation than noninjured peers [9]. This reduced social participation has negative effects on employment, health, and quality of life [26]. As the previous literature suggests that these challenges may extend to web-based communication via social media [2,3,13,18], it is important to understand how and where individuals with TBI may face challenges in social media use. As the first step in this line of work, we examined social media usage to understand how TBI may affect participation in social media, as well as patterns of use for those who engage in this form of web-based communication.

In this study, a majority (51/53, 96%) of participants with a history of TBI were reported using at least one social media platform. All participants in this study had access to the internet to complete the survey, and the proportion of social media users was higher in the TBI group than in previous work on TBI (eg, Baker-Sparr et al [3], who reported that 79%, 197/250, of internet users with TBI in their sample had at least one social media account). These findings suggest that social media use is ubiquitous among individuals with TBI, just as it is for individuals without a history of TBI.

In several ways, participants with TBI were congruent with NC participants in their use of social media. The groups had the same most popular social media platforms (Facebook and Facebook Messenger). These popular platforms were also consistent with previous studies of individuals with TBI (eg, Baker-Sparr et al [3] and Brunner et al [13]). Participants with and without a history of TBI were also similar in that they more frequently use social media passively (eg, to read the news feed) than actively (eg, to post new things). This observation was consistent with previous work [13], suggesting that individuals with TBI, like their noninjured peers, may be more likely to use social media to observe others than to actively participate in themselves. Both groups were also reported most frequently that they are Facebook friends with close friends and family members, suggesting that participants with TBI and NC participants both use social media more to foster existing interpersonal relationships than to actively seek new ones. Future work might consider how these patterns of social media use reflect neural activity in individuals with and without a history of TBI [4].

We also found that NC participants reported more frequently than participants with TBI that they use some platforms, including LinkedIn, Zoom, and Skype. Even participants with TBI who have accounts on these platforms report using them less frequently than NC participants. It is interesting to note that many of the platforms used more frequently by NC participants (eg, LinkedIn, Zoom, and Skype) are often used in a professional context. In fact, participants with TBI were more similar to NC participants in their use of FaceTime, another videoconferencing app that is not often used in a professional context. Individuals with TBI may be less likely to hold careers that depend on web-based communication or where web-based networking is critical to success. Although speculative, it is worth considering whether the relationship is bidirectional, with the challenges of CMC limiting the interest of or opportunities for adults with TBI in work that involves a great deal of web-based communication. In fact, our results were consistent with previous work [13], suggesting that few people with TBI use social media for professional networking or TBI-related advocacy. In today’s connected digital world, addressing this *digital divide* may allow individuals with TBI to increase their professional and personal self-advocacy on a broader scale [27].

Although both individuals with TBI and noninjured peers use social media more passively than actively, some individuals with TBI may be less likely than their noninjured peers to use CMC in a way that translates to in-person communication, relationships, and events happening beyond social media [6]. NC participants reported more frequently than participants with TBI that they use social media to keep up with friends and family or to get information regarding social events. NC participants also reported more frequently that they use social media for more distant networking opportunities. NC participants reported more frequently than participants with TBI that they are Facebook friends with acquaintances, coworkers, or people they had met once or casually dated. Consistent with previous work [18], individuals with TBI may not use social media as an alternative to in-person communication but rather face similar challenges in web-based and offline communication that may prevent them from capitalizing on the benefits of social media. Intervening in CMC, just as in-person communication, may allow individuals with TBI to increase their active participation in web-based activities that may translate to relationships and reduce social isolation beyond social media.

#### Social Media Presents Challenges and Opportunities for Adults With TBI

Many of the self-reported group differences in patterns of social media use in this study may be driven by a subset (12/53, 23%) of participants with TBI who reported changes in their social media use caused by TBI. This subgroup of individuals who readily identify injury-related social media changes is consistent with considerable heterogeneity in cognitive and disability profiles for individuals with a history of brain injury [10]. For example, it is estimated that approximately one-third of individuals with TBI exhibit social cognition deficits [28]. It is important to note that cognitive impairment (eg, in memory or social communication) may not always result in a functional disability that affects social media use, especially for individuals who develop or use adaptive strategies.

As with any intervention targeting the heterogeneous group of individuals with a history of TBI, it is likely that a critical part of a successful social media intervention will be identifying those individuals who will benefit. Future work should consider whether, and if so how, the subgroups of individuals with postinjury changes to social media use and social cognition overlap (ie, if the subgroup of participants who report social media changes are experiencing an extension of challenges present in offline social communication [18]). However, it is important to consider how cognitive-communication adaptations might support CMC use for some individuals with TBI who do not report a change in their use of social media, possibly because of a lack of insight or deficit awareness. Future intervention work should assess the efficacy of social media modifications in increasing CMC success for individuals with TBI who do and do not report changes to their social media use postinjury.

Research on social communication in adults with TBI has revealed impairments in perception of social cues even in face-to-face, synchronous communication [29-34]. In this study, participants with TBI reported less frequently than NC participants that they use some platforms involving synchronous communication (eg, Zoom and Skype), although they were more comparable in their use of FaceTime. This pattern was consistent with findings from a separate survey on the COVID-19 pandemic [35], in which individuals with a history of TBI reported that they found video chat to be less successful than face-to-face communication, and some participants stated that impoverished visual and verbal cues via video chat make it more difficult to read social signals. In this context, it is interesting to consider how asynchronous communication via social media may prove even more challenging for some individuals with TBI than synchronous video chat. Communicating successfully via text on social media (eg, via Facebook posts) requires the integration of a broad range of social and contextual multimedia cues, as well as considerable social inferencing, without any verbal information or the ability to read gestures or facial expressions. As such, it may be useful to support individuals with TBI in isolating important social cues (eg, keywords and emojis) available in communication over social media.

There are certainly risks to social media use, which were identified by some participants in our study. Some individuals with TBI may be more vulnerable to social media overuse, cyberbullying, or web-based manipulation because of disabilities in self-regulation, decision-making, and resiliency [2,13]. In an earlier study [13], individuals with TBI reported being bullied on the internet at a high rate. As such, rather than issuing a blanket recommendation that individuals with TBI use social media to increase their participation, it is critical to consider how to support individuals with TBI in using social media in a way that is beneficial, as well as filtering and responding to critical information when on the web [3,13].

At the same time, there is great opportunity to reduce barriers to social media use for individuals with TBI. Some preliminary work [18] has suggested that using social media helps individuals with TBI to increase their broader social participation. The increased movement for disability advocacy on the web presents an opportunity for adults with TBI to engage with others who have shared experiences [2,7,13], and several participants in this study identified increased opportunities for TBI engagement as an area for social media growth. Furthermore, supporting individuals with TBI in their use of social media may reduce the *digital divide* with regard to web-based professional networking and using social media in a way that translates to real-world professional and personal opportunities [27].

### Designing Tailored and Effective Social Media Supports

This study represents a critical step in developing technology-based social media interventions for individuals with TBI, as stakeholders should have a guiding voice in rehabilitation research [19]. Here, individuals with TBI exhibited social media usage patterns that are broadly similar to noninjured peers, with limited exceptions. However, a proportion of respondents identified barriers that affect their social media use. In the context of designing social media interventions, these findings raise the question of whether individuals with TBI receive the same benefits from social media use as noninjured peers. For example, it is possible that some individuals with TBI do not explore or interact with social media in the same way as their peers. It may be challenging to sample or recall information from visually complex, dynamic displays or to read and broadcast appropriate web-based social cues in a way that increases broader social capital. Future work should assess this open question to determine the critical places for technology-based interventions for social media use in TBI.

Evaluating the effectiveness of an intervention should consider not only communication success but also the accessibility, usefulness, and acceptance of that intervention for individuals with TBI [36]. As technology support is more likely to create meaningful positive changes when in regular use, it is critical to solicit and follow the guidance of individuals with TBI when considering where and how interventions can support successful social media use.

### Limitations to Generalizability

The results of this study provide a snapshot of how individuals with TBI use social media and their priorities for potential social media–based interventions. As we administered our survey on the web, all participants in this study had regular access to email, and thus, our sample may not be fully representative of the spectrum of internet use in TBI. Here, we present descriptive data that may form the foundation for future hypothesis-driven intervention research in this area [24]. Our participants provided initial responses as to how we might improve social media support for individuals with TBI, but further studies may build on these results to request feedback on specific intervention options. Additional studies with larger sample sizes will also allow for informed hypotheses and direct statistical tests of between-group differences in social media use.

### Conclusions

Consistent with previous work [2,3,13], cognitive disabilities may add to the social media maze for some individuals with TBI, and designing supports that mitigate these challenges may increase web-based and real-world social participation. Although it is possible that only a subset of individuals with TBI would benefit from technology-based social media support, to the extent that social participation on the web helps to reduce the physical and psychological burdens of loneliness, developing such interventions is warranted. As technology evolves, the principles of these interventions should be well-defined, evidence-based, and generalizable beyond a single platform. They should also reflect the priorities and input of individuals with TBI [19,36]. Furthermore, given the heterogeneity of social media patterns in this sample, interventions should be easily tailored to a given individual. By considering CMC as part of an individual’s broader social health, we stand to alter web-based participation in a way that is meaningful, positive, and beneficial to broader social life.

## Appendix

Multimedia Appendix 1 Survey questions and full frequency table.

## Abbreviations

CMC: computer-mediated communication
NC: noninjured comparison
REDCap: Research Electronic Data Capture
TBI: traumatic brain injury
