# Supplementary material for: Computer-Mediated Communication in Adults With and Without Moderate-to-Severe Traumatic Brain Injury: Survey of Social Media Use
Source: JMIR Rehabil Assist Technol. 2021 Aug 27;8(3):e26586. doi: 10.2196/26586 (PMC8433938; doi:10.2196/26586)
Supplement: Multimedia Appendix 1 [file rehab_v8i3e26586_app1.docx]

Supplementary Materials

1. **Survey questions for this dataset**
2. **Please check all the social media platforms where you currently have an account.**

**(Please check all that apply.)**

- Bumble
- Discord
- Facebook
- Facebook Messenger
- FaceTime
- Google Hangouts
- Hinge
- Instagram
- LINE
- LinkedIn
- Pinterest
- Quora
- Reddit
- Skype
- Snapchat
- Telegram
- TikTok
- Tinder
- Tumblr
- Twitter
- Viber
- WhatsApp
- Zoom
- Other platform, not listed here

1. (If checked “Other platform” in #1)

**Please list the other platforms (not listed above) where you have an account:** _______________

1. **Are there any social media platforms where you would like to have an account, but do not currently?**

- Yes
- No

1. (If answered “Yes” to #3)

**Please check any social media platforms where you would like to have an account, but do not currently.**

**(Please check all that apply.)**

- Bumble
- Discord
- Facebook
- Facebook Messenger
- FaceTime
- Google Hangouts
- Hinge
- Instagram
- LINE
- LinkedIn
- Pinterest
- Quora
- Reddit
- Skype
- Snapchat
- Telegram
- TikTok
- Tinder
- Tumblr
- Twitter
- Viber
- WhatsApp
- Zoom
- Other platform, not listed here

1. (If answered “Yes” to #3)

**Please list the other platforms (not listed above) where you would like to have an account but do not currently:** _______________

1. (If answered “Yes” to #3)

**Why don’t you use these platform(s) that you would like to use?** ____________

1. **Please indicate how often you use the following social media platform(s):**

(Participants responded for each platform they checked in #1.)

- - Yearly
  - Monthly
  - Weekly
  - Multiple times a week
  - Daily
  - Multiple times a day

1. **I use social media for ______________. (Please check all that apply.)**
   - Advocating for specific causes (e.g., promoting TBI-related organizations or events)
   - Creating my social identity
   - Discovering new things
   - Following thought leaders or celebrities
   - Getting information regarding social events
   - Getting job-related information
   - Keeping in touch with friends and family
   - Looking for support groups
   - Providing social support to others
   - Searching for specific information (e.g., information about TBI)
   - Staying up to date with news and current events
   - Sharing the happenings of daily life
   - Sharing new ideas
2. **Do you have a Facebook account?**
   - Yes
   - No
3. (If answered “No” to #9)

**Why not?** _________________

1. (For participants with TBI)

**Has your use of social media changed because of your TBI?**

**For example, some people might use social media more or less frequently after TBI. Others might choose to use different social media platforms after TBI.**

- Yes
- No

1. (If answered “Yes” to #11)

**How has your use of social media changed because of your TBI**? ____________

1. (For participants with TBI)

**Researchers and clinicians are interested in improving the experience of using social media for individuals with TBI.**

**Do you have any experiences or suggestions to share?** ___________________

1. (For Facebook users)

**Please indicate how often you do the following activities on Facebook:**

(Response choices: Never or almost never, Sometimes, Often)

- Post something
- Share thoughts and feelings
- Share something you are interested in
- Share your impressions with your friends
- Donate to a cause on Facebook
- Follow your friends’ news
- Look through your newsfeed
- Click on content shared by friends
- Browse your friends’ profiles
- Browse through friends of your friends
- Look at profiles of people not on your Facebook friends list
- Search for people to add
- Send friendship requests
- Add people suggested by Facebook
- Send private messages
- Chat

1. (For Facebook users)

**Please indicate which of the following are included in your Facebook friends.**

**(Please check all that apply.):**

- Acquaintance
- Band, musical artist, or other celebrity
- Best friend
- Classmate
- Coworker
- Current significant other (e.g., girlfriend or boyfriend)
- Family member
- Fellow club member
- Fraternity/sorority brother or sister
- Friend of a friend
- Good friend
- High school friend
- Neighbor
- Online friend only (never met in person)
- Past romantic partner
- Roommate
- Someone you do not know
- Someone you casually dated
- Someone you met in a different country
- Someone you only met once
- Teammate
- Very good friend
- Other

1. **Frequency table for all social media platforms.**

|  | **Yearly** | | **Monthly** | | **Weekly** | | **Multiple Times/Week** | | **Daily** | | **Multiple Times/Day** | |
| --- | --- | --- | --- | --- | --- | --- | --- | --- | --- | --- | --- | --- |
| **Platform** | **TBI** | **NC** | **TBI** | **NC** | **TBI** | **NC** | **TBI** | **NC** | **TBI** | **NC** | **TBI** | **NC** |
| Bumble  (TBI n= 7, NC n= 1) | 42.86% | 0% | 0% | 0% | 14.28% | 0% | 28.57% | 100% | 14.29% | 0% | 0% | 0% |
| Discord  (TBI n= 7, NC n= 4) | 0% | 25.00% | 75.00% | 50.00% | 0% | 0% | 0% | 25.00% | 0% | 0% | 25.00% | 0% |
| Facebook  (TBI n= 41, NC n= 43) | 0% | 4.65% | 2.44% | 9.30% | 12.19% | 2.33% | 9.76% | 16.28% | 29.27% | 20.93% | 46.34% | 46.51% |
| Facebook Messenger  (TBI n= 38, NC n= 40) | 5.26% | 10.00% | 7.90% | 17.50% | 15.79% | 20.00% | 26.32% | 27.50% | 23.68% | 12.50% | 21.05% | 12.50% |
| FaceTime  (TBI n= 25, NC n= 28) | 0% | 10.71% | 48.00% | 28.57% | 12.00% | 35.71% | 28.00% | 21.43% | 8.00% | 0% | 4.00% | 3.57% |
| Google Hangouts  (TBI n= 7, NC n= 12) | 42.86% | 33.33% | 42.86% | 25.00% | 0% | 16.67% | 14.28% | 0% | 0% | 8.33% | 0% | 16.67% |
| Hinge  (TBI n= 4, NC n= 4) | 50.00% | 0% | 0% | 25.00% | 25.00% | 25.00% | 25.00% | 50.00% | 0% | 0% | 0% | 0% |
| Instagram  (TBI n= 30, NC n= 37) | 6.67% | 2.70% | 16.67% | 5.41% | 10.00% | 2.70% | 6.67% | 10.81% | 13.33% | 32.43% | 46.66% | 45.95% |
| LINE  (TBI n= 0, NC n= 0) | - | - | - | - | - | - | - | - | - | - | - | - |
| LinkedIn  (TBI n= 19, NC n= 29) | 26.31% | 17.24% | 15.79% | 44.83% | 31.58% | 24.14% | 10.53% | 6.90% | 15.79% | 3.45% | 0% | 3.45% |
| Pinterest  (TBI n= 16, NC n= 23) | 31.25% | 26.09% | 25.00% | 34.78% | 12.50% | 13.04% | 18.75% | 17.39% | 6.25% | 0% | 6.25% | 8.70% |
| Quora  (TBI n= 0, NC n= 3) | - | 33.33% | - | 33.33% | - | 0% | - | 33.33% | - | 0% | - | 0% |
| Reddit  (TBI n= 8, NC n= 11) | 12.50% | 0% | 37.50% | 9.09% | 25.00% | 18.18% | 0% | 27.27% | 12.50% | 9.09% | 12.50% | 36.36% |
| Skype  (TBI n= 13, NC n= 23) | 46.15% | 30.43% | 30.77% | 34.78% | 0% | 8.70% | 7.69% | 4.35% | 15.39% | 13.04% | 0% | 8.70% |
| Snapchat  (TBI n= 25, NC n= 23) | 8.00% | 17.39% | 16.00% | 0% | 16.00% | 21.74% | 20.00% | 17.39% | 12.00% | 17.39% | 28.00% | 26.09% |
| Telegram  (TBI n= 1, NC n= 1) | 0% | 0% | 100% | 100% | 0% | 0% | 0% | 0% | 0% | 0% | 0% | 0% |
| TikTok  (TBI n= 3, NC n= 9) | 0% | 22.22% | 33.33% | 0% | 0% | 33.33% | 33.33% | 11.11% | 0% | 0% | 33.33% | 33.33% |
| Tinder  (TBI n= 6, NC n= 3) | 50.00% | 0% | 0% | 33.33% | 16.67% | 33.33% | 16.67% | 33.33% | 16.67% | 0% | 0% | 0% |
| Tumblr  (TBI n= 3, NC n= 7) | 33.33% | 28.57% | 33.33% | 42.86% | 0% | 14.29% | 0% | 0% | 0% | 14.29% | 33.33% | 0% |
| Twitter  (TBI n= 13, NC n= 22) | 23.08% | 13.64% | 15.39% | 27.27% | 15.38% | 13.64% | 23.08% | 9.09% | 7.69% | 13.64% | 15.38% | 22.73% |
| Viber  (TBI n= 0, NC n= 2) | - | 0% | - | 0% | - | 50.00% | - | 50.00% | - | 0% | - | 0% |
| WhatsApp  (TBI n= 15, NC n= 18) | 33.33% | 38.89% | 33.33% | 27.78% | 20.00% | 11.11% | 0% | 5.56% | 13.34% | 5.56% | 0% | 11.11% |
| Zoom  (TBI n= 19, NC n= 31) | 10.53% | 3.23% | 31.57% | 9.68% | 21.05% | 29.03% | 10.53% | 29.03% | 15.79% | 19.35% | 10.53% | 9.68% |
